# Long-term progression-free survival with first-line pyrotinib monotherapy in a treatment-naïve stage IV NSCLC patient harboring an ERBB2 exon 20 insertion: a case report

**DOI:** 10.3389/fonc.2025.1637973

**Published:** 2025-12-05

**Authors:** Zhenyu Ke, Hongbo Yang, Meng Xing, Shasha Bi, Fujun Yang

**Affiliations:** 1 Department of Oncology, Weihai Municipal Hospital, Cheeloo College of Medicine, Shandong University, Weihai, China; 2 Department of Pathology, Weihai Municipal Hospital, Cheeloo College of Medicine, Shandong University, Weihai, China

**Keywords:** non-small cell lung cancer, HER2 mutation, ERBB2 exon 20 insertion, Y772_A775dup, pyrotinib, first-line therapy, case report

## Abstract

HER2 (ERBB2) mutations, particularly exon 20 insertions, are rare but actionable oncogenic drivers in non-small cell lung cancer (NSCLC). Pyrotinib, an oral irreversible pan-ErbB tyrosine kinase inhibitor, has shown promising efficacy in previously treated HER2-mutant NSCLC, but its role in the first-line setting remains unclear. We report a case of a 61-year-old woman with stage IVB lung adenocarcinoma harboring an ERBB2 exon 20 insertion (p.Y772_A775dup) and a concurrent EGFR mutation, who was treated with first-line pyrotinib monotherapy. She achieved a partial response within one month and has maintained disease control for over 31 months, with only mild intermittent diarrhea. This case provides real-world evidence supporting the potential of pyrotinib as an effective first-line treatment for HER2-mutant NSCLC, particularly in patients with the Y772_A775dup variant and concurrent EGFR alterations, and highlights the need for further clinical investigation in this setting.

## Introduction

Non-small cell lung cancer (NSCLC) remains the leading cause of cancer-related mortality worldwide, with adenocarcinoma being its most common histological subtype (1). In recent years, advances in molecular profiling have identified a variety of oncogenic driver mutations that enable personalized treatment strategies. Among them, ERBB2 (also known as HER2) alterations have emerged as important targets, particularly in adenocarcinomas without EGFR, ALK, or ROS1 aberrations (2). ERBB2 exon 20 insertions represent the predominant subtype of ERBB2 mutations in NSCLC, accounting for approximately 2%–4% of cases. These mutations, especially the p.Y772_A775dup variant, lead to constitutive activation of the HER2 tyrosine kinase domain, promoting tumor proliferation and survival (3, 4). Unlike in breast and gastric cancers, where HER2 amplification and overexpression guide therapy, ERBB2-mutant NSCLC poses unique therapeutic challenges due to the structural nature and lower immunogenicity of the mutations.

Pyrotinib is an irreversible pan-ErbB tyrosine kinase inhibitor (TKI) targeting EGFR, HER2, and HER4 (5). It has shown promising antitumor activity in ERBB2-mutant NSCLC, particularly in those with exon 20 insertion mutations (6). In a phase II trial involving previously platinum-treated patients, pyrotinib monotherapy achieved an objective response rate (ORR) of 30%, with a median progression-free survival (mPFS) of 6.9 months and a median overall survival of 14.4 months (7). Subgroup analyses further indicated that its efficacy was independent of brain metastasis status or mutation subtype (exon 20 *vs*. non-exon 20). Notably, clinical benefit was also observed in patients who had received three or more prior lines of therapy (7). Although its role in second-line settings remains to be fully defined, current evidence supports pyrotinib as a viable targeted option for HER2-mutant NSCLC, including in those with brain metastases. However, data on its use as first-line monotherapy are still limited. Here, we report a rare case of advanced/metastatic NSCLC with an ERBB2 exon 20 insertion (p.Y772_A775dup) treated with first-line pyrotinib monotherapy, resulting in a durable response and a PFS exceeding 31 months. This case may provide clinical evidence supporting pyrotinib as a potential alternative first-line treatment for patients with HER2-mutant NSCLC.

## Case presentation

**Figure 1:**
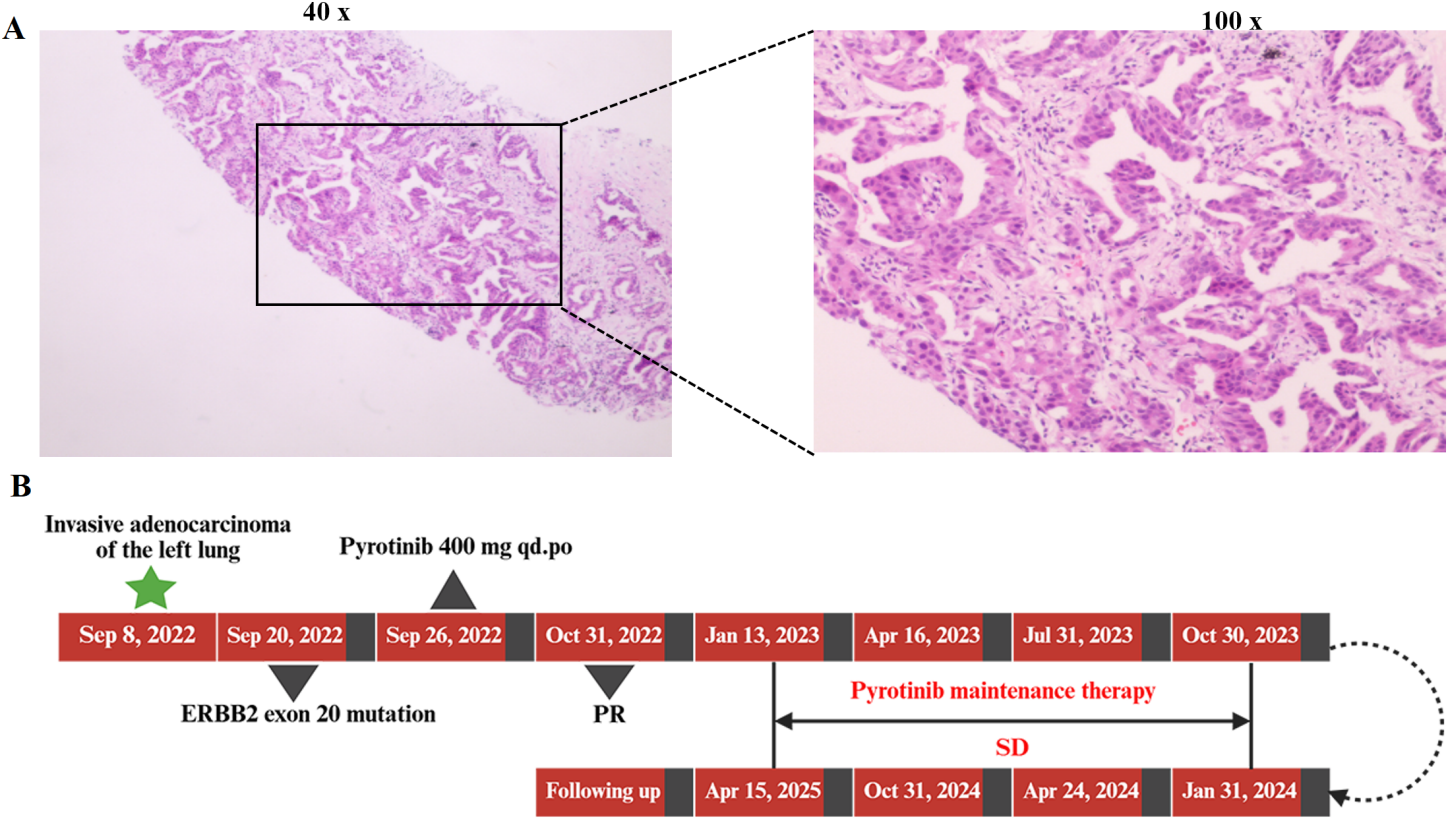
**(A)** Hematoxylin and eosin (H&E) staining of the biopsy specimen from the left lung lesion. **(B)** Timeline of diagnosis, treatment, and outcome assessment in this case.

A 61-year-old Han Chinese woman presented to the Department of Minimally Invasive Oncology on September 5, 2022, with a three-month history of paroxysmal cough and a two-day history of a newly detected left lung mass. She had no history of smoking and no known family history of malignancy. The patient had a 5-year history of hypertension, with a maximum blood pressure of 150/100 mmHg. Her blood pressure is currently well controlled at approximately 130/85 mmHg with sustained-release nifedipine (20 mg, twice daily). Contrast-enhanced chest CT revealed an irregular nodule in the apicoposterior segment of the left upper lobe, measuring approximately 1.6 cm × 2.6 cm in maximal cross-section. The lesion had spiculated margins with adjacent pleural retraction and a vascular convergence sign, and it showed enhancement after contrast administration. Multiple micro- and small nodules with clear borders were observed in both lungs and the left interlobar pleura. Enhanced scans demonstrated multiple enlarged lymph nodes in the mediastinum and left hilar region, suggesting metastases. High-density lesions were also seen in portions of the thoracic vertebral bodies, their appendages, and parts of the left ribs, highly suggestive of metastatic involvement. Bone ECT demonstrated increased bone metabolic activity in the left sides of the T7 and T9 vertebral bodies, the left posterior 7th and 8th ribs, and the left side of the L4 vertebral body. Partial lesions could not exclude the possibility of tumor bone metastases. Abdominal ultrasound and contrast-enhanced brain MRI showed no abnormalities. Serum tumor marker evaluation showed elevated levels of carcinoembryonic antigen (CEA) at 18.68 ng/mL, carbohydrate antigen 125 (CA125) at 56.78 U/mL, and cytokeratin fragment 21-1 (CYFRA21-1) at 4.7 ng/mL. On September 7, 2022, a CT-guided percutaneous biopsy of the left lung lesion was performed. Histopathological analysis on September 8 confirmed a moderately differentiated invasive adenocarcinoma of the left upper lobe (**Figure 1A**). The clinical staging was determined as cT4N2M1c, stage IVB, with metastases to bilateral lungs, the left interlobar pleura, mediastinal and left hilar lymph nodes, and the skeleton.

**Figure 2:**
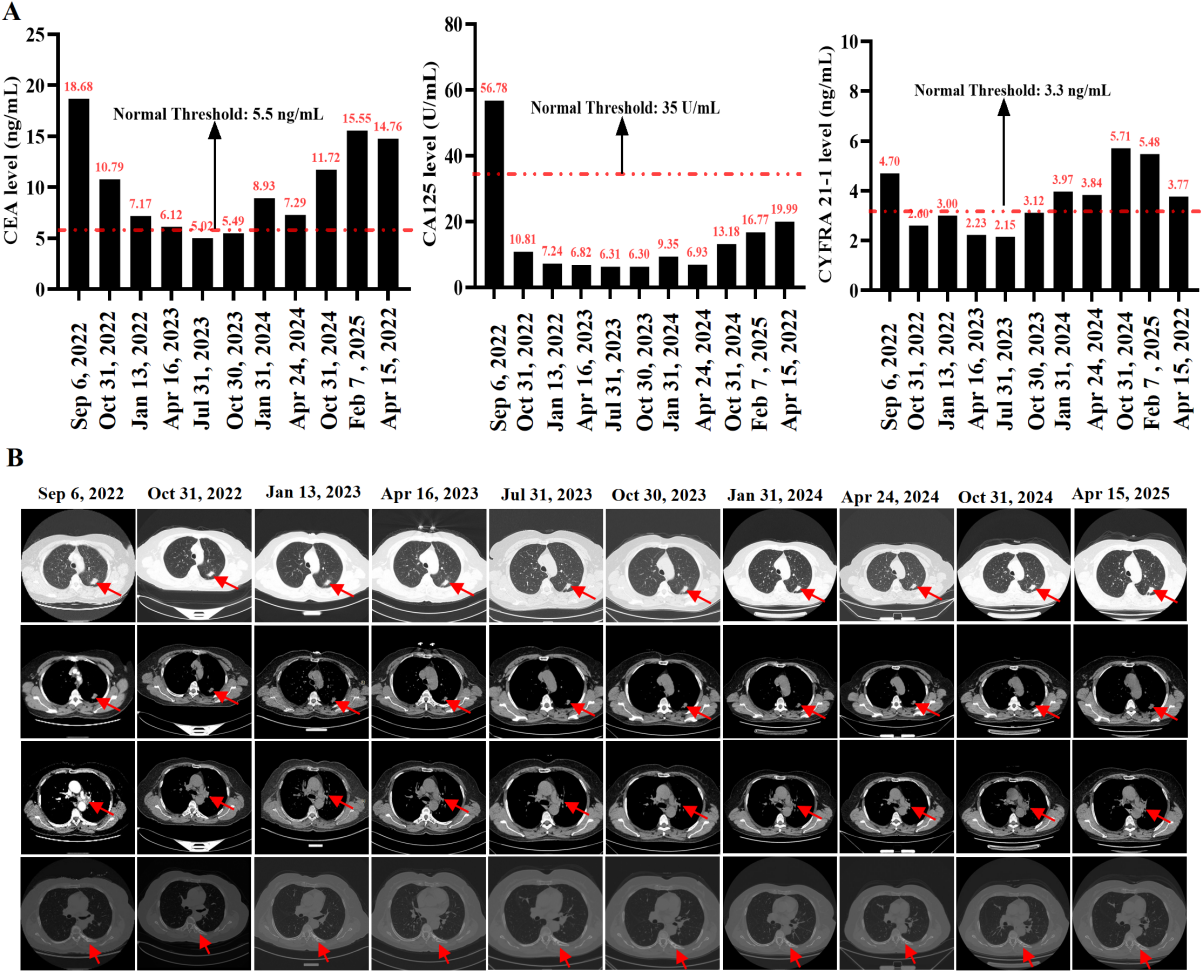
**(A)** Serial measurements of serum tumor markers including carcinoembryonic antigen (CEA), carbohydrate antigen 125 (CA125), and cytokeratin-19 fragment (CYFRA21-1) from baseline to April 15, 2025. **(B)** Representative serial chest computed tomography (CT) images from baseline (September 6, 2022) through follow-up (April 15, 2025). Red arrows indicate the primary lung lesion.

Next-generation sequencing (NGS) using the MGISEQ-200 platform developed by BGI was performed on tumor tissue samples from the patient with a targeted panel covering 50 non–small-cell lung cancer–related genes, including both exonic and selected intronic regions. DNA extraction met quality requirements (≥100 ng), and sequencing achieved a mean depth of 2276.77×, with 100% coverage at 1× and 99.63% coverage at 100× across the target regions. The Q30 base quality ratio was 89.71%, all exceeding the preset quality control thresholds (≥500×, ≥99%, ≥95%, and ≥80%, respectively). NGS results revealed an ERBB2 p.Y772_A775dup (exon 20) mutation with a variant abundance of 30.91% (Class I), along with multiple Class III variants in genes including *EGFR* (p.T1029M, p.R962H, p.A10V), *TP53* (p.I505S), *U2AF1* (p.S34F), *KEAP1* (p.L81Q), *SMAD4* (p.H262Q), and *KIT* (p.K807N, p.I808L) (**Table 1**). Although the *EGFR* p.T1029M (c.3086C>T) and *EGFR* p.R962H (c.2885G>A) variants represent alterations within the *EGFR* gene, they have not been widely recognized as canonical resistance mutations. At present, their clinical significance remains uncertain, and their potential association with resistance to targeted therapies has yet to be definitively established. Therefore, these variants are classified as Class III variants. Following a multidisciplinary team (MDT) discussion involving medical oncology, radiation oncology, and thoracic surgery, the patient was offered three potential treatment options (1): bevacizumab combined with platinum-based doublet chemotherapy, with or without local radiotherapy (standard first-line therapy); (2) platinum-based chemotherapy combined with immunotherapy, with or without local radiotherapy; or (3) pyrotinib monotherapy as an individualized treatment, with or without local radiotherapy. Considering the patient’s general condition and tolerance, chemotherapy was declined. Based on the findings of a multicenter, open-label, single-arm phase II clinical study evaluating the efficacy and safety of pyrotinib as a second-line or later treatment for advanced NSCLC with *HER2* mutations, and given that the patient’s genetic testing revealed an *ERBB2* exon 20 insertion mutation, The patient and her family ultimately chose oral pyrotinib monotherapy (400 mg once daily) as an individualized regimen, and treatment was initiated on September 26, 2022 (**Figure 1B**). A follow-up evaluation on October 31 showed a significant reduction in the size of the primary lesion, with the target lesion measuring 1.8 cm × 1.1 cm one month after treatment. In addition, the mediastinal lymph nodes had decreased in size, and the bone metastases remained stable. Tumor markers also decreased markedly one month after treatment (CEA, 10.79 ng/mL; CA125, 10.81 U/mL; CYFRA21-1, 2.6 ng/mL) (**Figure 2A**). The clinical response was assessed as partial response (PR) (**Figure 2B**). Although local radiotherapy was recommended again at that time, the patient and her family declined and chose to continue with pyrotinib maintenance therapy. As of the most recent evaluation on April 15, 2025, the patient maintained stable disease (SD) without any evidence of progression. The patient’s ECOG performance status remained at 1, and she reported intermittent cough without sputum production. Diarrhea developed three days after the initiation of oral pyrotinib, occurring two to three times per day and characterized by yellow, watery stools. According to the CTCAE criteria, this event was classified as Grade 1. The condition was managed with self-administered loperamide, resulting in symptom relief, and no dose adjustment of pyrotinib was required. No other adverse events, such as nausea, vomiting, rash, paronychia, hand-foot syndrome, or abnormal liver function, were observed during the treatment period.

**Table 1:** Genetic variant test results obtained through next-generation sequencing.

Gene name	Test result	Gene region	Mutation abundance	Variant classification
ERBB2	p.Y772_A775dup (c.2313_2324dupATACGTGATGGC)	EX20	30.91%	I
U2AF1	p.S34F (c.101C>T)	EX2	16.81%	III
EGFR	p.T1029M (c.3086C>T)	EX25	46.39%	III
EGFR	p.R962H (c.2885G>A)	EX24	45.01%	III
TP53	p.I505S (c.149T>G)	EX4	2.97%	III
KEAP1	p.L81Q (c.242T>A)	EX2	2.45%	III
SMAD4	p.H262Q (c.786T>A)	EX6	2.40%	III
KIT	p.K807N (c.2421G>T)	EX17	2.03%	III
KIT	p.I808L (c.2422A>C)	EX17	1.96%	III
EGFR	p.A10V	EX1	0.85%	III

## Discussion

NSCLC accounts for over 85% of all lung cancer cases and remains a leading cause of cancer-related mortality worldwide (8). While the advent of targeted therapies has significantly improved outcomes in patients with driver mutations such as EGFR, ALK, and KRAS, effective treatments for HER2 (ERBB2)-mutant NSCLC remain limited (9). HER2 mutations, particularly exon 20 insertions, occur in approximately 2%–3% of NSCLC cases and are generally associated with poor prognosis and limited response to standard chemotherapy or immunotherapy (10). Moreover, HER2 amplification is a recognized mechanism of acquired resistance to EGFR inhibitors (11). These alterations can activate oncogenic pathways such as PI3K-AKT-mTOR and MAPK, promoting tumor growth (12). Although HER2-directed therapies, including antibody-drug conjugates and TKIs, have demonstrated modest efficacy, overall response rates typically range from 30% to 50%, with a mPFS of only 4–6 months. The limited durability of these responses is often attributed to diverse resistance mechanisms, underscoring the need for more effective strategies.

Pyrotinib is an oral, irreversible pan-ErbB TKI independently developed by Jiangsu Hengrui Pharmaceuticals in China. It was approved by the National Medical Products Administration (NMPA) in 2018 for use in combination with capecitabine in patients with HER2-positive advanced or metastatic breast cancer, particularly those who have previously received HER2-targeted therapies (13). In addition, pyrotinib has demonstrated promising clinical activity in NSCLC patients harboring HER2 mutations, especially exon 20 insertions (13). Although it has not yet been officially approved by the NMPA for the treatment of NSCLC, multiple phase II and III clinical studies have shown ORR ranging from 20% to 30%, with a favorable safety profile. As a result, pyrotinib has been widely adopted in China as an individualized targeted treatment for HER2-mutant NSCLC, including in second-line and exploratory first-line settings. Studies have reported a median PFS ranging from 9.87 months to 15.0 months for pyrotinib monotherapy or combination regimens in patients with advanced lung adenocarcinoma, outperforming traditional chemotherapy (5.40 months). Additionally, in HER2-amplified NSCLC patients, first-line pyrotinib treatment achieved an objective response rate of 35.7% and a disease control rate (DCR) of 89.3% (14). Some case reports suggest that for patients with non-Tyrosine kinase domain ERBB2 mutations, pyrotinib monotherapy as first-line treatment can achieve partial response or complete response (15, 16). However, the current evidence supporting the use of pyrotinib as a first-line treatment for NSCLC is primarily derived from retrospective studies, small-scale clinical trials, and case reports. Research in this area remains in its early stages, and there is still a lack of large-scale prospective studies.

Although previous studies have shown that responses to pyrotinib were observed across different HER2 mutation subtypes—including both exon 20 and non-exon 20 variants—with disease control rates (DCR) ranging from 81.8% to 100% (6), emerging evidence suggests that the sensitivity of HER2-mutant NSCLC to pyrotinib may vary depending on the specific mutation subtype. For instance, a case report described a patient with a HER2 M774delinsWLV insertion who experienced rapid disease progression after three months of first-line pyrotinib monotherapy; however, subsequent combination therapy with the antiangiogenic agent bevacizumab extended PFS to 11 months (17). In contrast, another case involving a patient with a rare HER2 p.R456C mutation achieved a PFS of 13 months with pyrotinib monotherapy, suggesting that certain mutation sites may enhance drug-binding affinity or inhibit downstream signaling more effectively (18). Furthermore, Wu et al. reported that among exon 20 mutation subtypes, the Y772_A775 duplication was associated with the most favorable survival outcomes, with a median duration of response of 10.7 months and a median PFS of 7.3 months in a cohort of 22 patients. Interestingly, the patient with Y772_A775 duplication reported in this study has achieved a PFS of over 31 months following first-line pyrotinib monotherapy.

EGFR mutations such as exon 19 deletions and L858R are the most common driver alterations in NSCLC, and EGFR-TKIs are the standard first-line therapy. In contrast, ERBB2 alterations, including amplification or exon 20 insertions, have been recognized as emerging therapeutic targets, particularly in EGFR wild-type or EGFR-TKI–resistant tumors (19, 20). Co-mutations of EGFR and ERBB2 may contribute to complex molecular mechanisms and distinct treatment responses. Studies have shown that such co-alterations can reduce sensitivity to EGFR-TKIs and promote resistance through synergistic activation of EGFR/ERBB2 signaling, stabilizing downstream PI3K/AKT and RAS/RAF/MEK/ERK pathways (21–23). Additionally, ERBB2 exon 20 and EGFR exon 20 insertion mutations have been associated with an immunosuppressive tumor microenvironment (24). However, patients harboring both EGFR and ERBB2 alterations—such as co-occurrence of EGFR-sensitive mutations with ERBB2 amplification or exon 20 insertions—may benefit from pyrotinib due to its dual inhibition of both signaling pathways, potentially producing a synergistic antitumor effect (25). For example, one report described sustained tumor shrinkage and long-term survival in a patient with concurrent EGFR mutation and HER2 amplification who was treated with a combination of osimertinib and pyrotinib. Consistent with these findings, the present case describes a patient with co-occurring ERBB2 exon 20 Y772_A775dup and EGFR mutation who achieved a PFS exceeding 31 months following first-line pyrotinib monotherapy, highlighting the potential clinical benefit of this approach in selected molecular context.

Although pyrotinib has not been approved by the U.S. Food and Drug Administration (FDA) or the European Medicines Agency (EMA), and its global applicability remains to be established, it has shown encouraging therapeutic potential in HER2-mutant NSCLC. Most existing data are derived from phase II or small-scale studies, and its clinical value awaits confirmation from ongoing phase III trials. In this case, first-line pyrotinib monotherapy achieved a progression-free survival exceeding 31 months with good tolerability—one of the longest durations reported to date. The presence of a concurrent EGFR mutation may have contributed to enhanced efficacy, suggesting possible synergistic effects. This case provides valuable real-world evidence supporting further investigation of pyrotinib as a personalized treatment strategy in HER2-driven NSCLC, particularly in patients harboring the ERBB2 exon 20 Y772_A775dup mutation.

In clinical practice, pyrotinib is often administered in combination with chemotherapeutic agents such as capecitabine, paclitaxel, or gemcitabine, which has been shown to significantly improve the objective response rate and PFS (26). This benefit is particularly evident in patients who have experienced disease progression after prior anti-HER2 therapies (e.g., trastuzumab or lapatinib) (27). The most common adverse events associated with pyrotinib combination therapy include diarrhea, rash, hand-foot syndrome, myelosuppression, and abnormal liver function. Among these, diarrhea is the most frequent dose-limiting toxicity, with an incidence exceeding 70% (28–30). Most cases present as mild to moderate diarrhea, which can be effectively managed with symptomatic treatment; however, severe cases may require dose adjustment or temporary discontinuation of pyrotinib (31, 32). Myelosuppression, characterized by leukopenia, anemia, and thrombocytopenia, is more common when pyrotinib is combined with chemotherapy, increasing the risk of infection (13). Cardiovascular adverse events, including hypertension, arrhythmia, atrial fibrillation, and heart failure (32), have also been reported and require close clinical observation. Compared with combination regimens, pyrotinib monotherapy has gained increasing attention in recent years due to its favorable safety profile and lower toxicity, making it a more suitable option for elderly or treatment-intolerant patients. The main adverse events of monotherapy include diarrhea, nausea, vomiting, and hematologic toxicities such as leukopenia and anemia (33). Diarrhea remains the most frequent event, but most cases are Grade 1–2 and can be well controlled with symptomatic management. Severe adverse events (≥Grade 3) are rare, and no treatment-related deaths have been reported. Real-world data further confirm that diarrhea is the most common but generally mild adverse effect of pyrotinib, while myelosuppression and hepatic dysfunction are infrequent and manageable, thereby ensuring good treatment tolerability (31). Consistent with these findings, this patient experienced only mild Grade 1 diarrhea during pyrotinib therapy, without nausea, vomiting, rash, paronychia, hand-foot syndrome, or hepatic dysfunction.

Despite this case report provides valuable insights into the potential of pyrotinib as a first-line treatment for HER2-mutant NSCLC, but several limitations should be acknowledged. First, the study is based on a single patient, limiting the generalizability of the findings. Larger clinical trials are needed to confirm pyrotinib’s efficacy and safety, especially in patients with the Y772_A775dup mutation and concurrent EGFR alterations. Second, the observed long-term PFS may not be representative, as individual responses to treatment can vary. While pyrotinib showed a favorable safety profile in this case, long-term toxicity and adverse effects need further validation in larger cohorts. Third, the co-occurring EGFR mutation may have contributed to the clinical benefit, suggesting a potential synergistic effect. Further investigation is required to explore the impact of combining pyrotinib with EGFR inhibitors. Finally, molecular profiling from other metastatic sites, such as brain metastases, was not included, which would provide a more comprehensive understanding of pyrotinib’s activity. Future studies should address these limitations and involve larger, more diverse populations to better establish pyrotinib’s role in HER2-mutant NSCLC treatment.

## Data Availability

The original contributions presented in the study are included in the article/supplementary material. Further inquiries can be directed to the corresponding authors.

## References

[1] Remon J Soria JC Peters S . Early and locally advanced non-small-cell lung cancer: an update of the ESMO Clinical Practice Guidelines focusing on diagnosis, staging, systemic and local therapy. Ann Oncol. (2021) . 32:1637–42. doi: 10.1016/j.annonc.2021.08.1994, PMID: 34481037

[2] Stephens P Hunter C Bignell G Edkins S Davies H Teague J . Lung cancer: intragenic ERBB2 kinase mutations in tumours. Nature. (2004) . 431:525–6. doi: 10.1038/431525b, PMID: 15457249

[3] Arcila ME Chaft JE Nafa K Roy-Chowdhuri S Lau C Zaidinski M . Prevalence, clinicopathologic associations, and molecular spectrum of ERBB2 (HER2) tyrosine kinase mutations in lung adenocarcinomas. Clin Cancer Res. (2012) . 18:4910–8. doi: 10.1158/1078-0432.ccr-12-0912, PMID: 22761469 PMC3865806

[4] Mazières J Peters S Lepage B Cortot AB Barlesi F Beau-Faller M . Lung cancer that harbors an HER2 mutation: epidemiologic characteristics and therapeutic perspectives. J Clin Oncol. (2013) . 31:1997–2003. doi: 10.1200/jco.2012.45.6095, PMID: 23610105

[5] Blair HA . Pyrotinib: first global approval. Drugs. (2018) . 78:1751–5. doi: 10.1007/s40265-018-0997-0, PMID: 30341682

[6] Liu SM Tu HY Wei XW Yan HH Dong XR Cui JW . First-line pyrotinib in advanced HER2-mutant non-small-cell lung cancer: a patient-centric phase 2 trial. Nat Med. (2023) . 29:2079–86. doi: 10.1038/s41591-023-02461-x, PMID: 37488286

[7] Zhou C Li X Wang Q Gao G Zhang Y Chen J . Pyrotinib in HER2-mutant advanced lung adenocarcinoma after platinum-based chemotherapy: A multicenter, open-label, single-arm, phase II study. J Clin Oncol. (2020) . 38:2753–61. doi: 10.1200/jco.20.00297, PMID: 32614698

[8] Florez N Patel SP Wakelee H Bazhenova L Massarelli E Salgia R . Proceedings of the 1st biannual bridging the gaps in lung cancer conference. Oncologist. (2025) 30:oyae228. doi: 10.1093/oncolo/oyae228, PMID: 39237103 PMC11883156

[9] Wang L Wu Y Ren Z Chu X Chen J Liu L . A retrospective study of first-line therapy and subsequent pyrotinib treatment in advanced lung adenocarcinoma with HER2 mutations. Cancer Med. (2024) . 13:e7335. doi: 10.1002/cam4.7335, PMID: 38923311 PMC11194746

[10] Pan X Zhou X . Long term survival achieved through combination of almonertinib and pyrotinib in EGFR-mutant/HER2-amplified advanced NSCLC patient: a case report and literature review. Front Oncol. (2024) . 14:1397238. doi: 10.3389/fonc.2024.1397238, PMID: 39184039 PMC11341367

[11] Han Y Xiong Y Lu T Chen R Liu Y Tang H . Genomic landscape and efficacy of HER2-targeted therapy in patients with HER2-mutant non-small cell lung cancer. Front Oncol. (2023) . 13:1121708. doi: 10.3389/fonc.2023.1121708, PMID: 37077822 PMC10106648

[12] Mao S Liu X Wang L Wang Y Yang S Jiang T . AYVM to AYMM transition on HER2 exon 20 insertion induces tyrosine kinase inhibitor resistance in NSCLC. J Thorac Oncol. (2025) . 20:739–49. doi: 10.1016/j.jtho.2024.12.022, PMID: 39725168

[13] Xu B Yan M Ma F Hu X Feng J Ouyang Q . Pyrotinib plus capecitabine versus lapatinib plus capecitabine for the treatment of HER2-positive metastatic breast cancer (PHOEBE): a multicentre, open-label, randomised, controlled, phase 3 trial. Lancet Oncol. (2021) . 22:351–60. doi: 10.1016/s1470-2045(20)30702-6, PMID: 33581774

[14] Song Z Lv D Chen SQ Huang J Li Y Ying S . Pyrotinib in patients with HER2-amplified advanced non-small cell lung cancer: A prospective, multicenter, single-arm trial. Clin Cancer Res. (2022) . 28:461–7. doi: 10.1158/1078-0432.ccr-21-2936, PMID: 34753778

[15] Ni J Si XY Zhang L . Non-small-cell lung cancer with ERBB2 mutation in non-tyrosine kinase domain benefits from pyrotinib: A case report. Thorac cancer. (2021) . 12:1244–7. doi: 10.1111/1759-7714.13889, PMID: 33655632 PMC8046093

[16] Gong K Yang Y Huang H Kuang X Yang X . HER2-amplified metastatic lung adenocarcinoma responds to fourth-line pyrotinib therapy: A case report. Mol Clin Oncol. (2021) . 15:213. doi: 10.3892/mco.2021.2375, PMID: 34476097 PMC8408680

[17] Yang G Liu R Tang X . Dacomitinib exhibits promising activity against the rare HER2 exon 20 insertion M774delinsWLV in lung cancer: A case report and literature review. Heliyon. (2024) . 10:e30312. doi: 10.1016/j.heliyon.2024.e30312, PMID: 38707278 PMC11068806

[18] Wang Y Hu J Liu R Li P Wang L Yang G . Promising response to pyrotinib in non-small-cell lung cancer with the rare HER2 R456C mutation: A case report. Curr Cancer Drug Targets. (2025). doi: 10.2174/0115680096371951250409093625, PMID: 40357782

[19] Ismail A Desai A Boumber Y . HER2 alterations in non-small cell lung cancer (NSCLC): from biology and testing to advances in treatment modalities. Front Oncol. (2025) . 15:1624124. doi: 10.3389/fonc.2025.1624124, PMID: 40620714 PMC12226463

[20] Hong L Patel S Drusbosky LM Xiong Y Chen R Geng R . Molecular landscape of ERBB2 alterations in 3000 advanced NSCLC patients. NPJ Precis Oncol. (2024) . 8:217. doi: 10.1038/s41698-024-00720-9, PMID: 39354054 PMC11445497

[21] Gan J Huang Y Liao J Pang L Fang W . HER2 amplification in advanced NSCLC patients after progression on EGFR-TKI and clinical response to EGFR-TKI plus pyrotinib combination therapy. Onco Targets Ther. (2021) . 14:5297–307. doi: 10.2147/ott.s335217, PMID: 34824536 PMC8609241

[22] Wei XW Deng JY Xu CR Chen ZH Zhu DQ Wu Q . Characteristics of and treatment strategies for advanced EGFR-mutant NSCLC with concomitant BRAF variations. JTO Clin Res Rep. (2022) . 3:100348. doi: 10.1016/j.jtocrr.2022.100348, PMID: 35789792 PMC9250018

[23] Ma S Zhang L Ren Y Dai W Chen T Luo L . Epiregulin confers EGFR-TKI resistance via EGFR/ErbB2 heterodimer in non-small cell lung cancer. Oncogene. (2021) . 40:2596–609. doi: 10.1038/s41388-021-01734-4, PMID: 33750895

[24] Kirchner M Kluck K Brandt R Volckmar AL Penzel R Kazdal D . The immune microenvironment in EGFR- and ERBB2-mutated lung adenocarcinoma. ESMO Open. (2021) . 6:100253. doi: 10.1016/j.esmoop.2021.100253, PMID: 34487971 PMC8426209

[25] Chen K Li W Xi X Zhong J . A case of multiple primary lung adenocarcinoma with a CD74-NRG1 fusion protein and HER2 mutation benefit from combined target therapy. Thorac cancer. (2022) . 13:3063–7. doi: 10.1111/1759-7714.14636, PMID: 36096509 PMC9626339

[26] Hu W Yang J Zhang Z Xu D Li N . Pyrotinib for HER2-positive metastatic breast cancer: a systematic review and meta-analysis. Trans Cancer Res. (2023) . 12:247–56. doi: 10.21037/tcr-22-1746, PMID: 36915587 PMC10007886

[27] Li C Bian X Liu Z Wang X Song X Zhao W . Effectiveness and safety of pyrotinib-based therapy in patients with HER2-positive metastatic breast cancer: A real-world retrospective study. Cancer Med. (2021) . 10:8352–64. doi: 10.1002/cam4.4335, PMID: 34672424 PMC8633258

[28] Dai L Gao T Guo R Chen Y Wang J Zhou S . Efficacy and safety of pyrotinib-based regimens in HER2 positive metastatic breast cancer: A retrospective real-world data study. Neoplasia. (2024) . 56:101029. doi: 10.1016/j.neo.2024.101029, PMID: 39024777 PMC11305273

[29] Tian C Wang M Liu H Liu J Xu M Ma L . Efficacy and safety of neoadjuvant pyrotinib plus docetaxel/liposomal doxorubicin/cyclophosphamide for HER2-positive breast cancer. Ir. J Med Sci. (2023) . 192:1041–9. doi: 10.1007/s11845-022-03093-9, PMID: 35829909

[30] Lin X Liu X Yang X Sun F . Efficacy and safety of neoadjuvant pyrotinib for human epidermal receptor 2-positive breast cancer: A meta-analysis. Tohoku J Exp Med. (2024) . 263:175–84. doi: 10.1620/tjem.2024.J026, PMID: 38658346

[31] Li Y Tong Z Wu X Ouyang Q Cai L Li W . Real-world treatment patterns and outcomes of pyrotinib-based therapy in patients with HER2-positive advanced breast cancer (PRETTY): A nationwide, prospective, observational study. Int J Cancer. (2023) . 153:1809–18. doi: 10.1002/ijc.34676, PMID: 37543965

[32] Yang G Xu H Yang Y Zhang S Xu F Hao X . Pyrotinib combined with apatinib for targeting metastatic non-small cell lung cancer with HER2 alterations: a prospective, open-label, single-arm phase 2 study (PATHER2). BMC Med. (2022) . 20:277. doi: 10.1186/s12916-022-02470-6, PMID: 36031613 PMC9422117

[33] Yang Z Fu WD Gu HY Ding JL Guo GL . A retrospective real-world study of pyrotinib in HER-2 positive advanced breast cancer. Cancer Manage Res. (2025) . 17:441–60. doi: 10.2147/cmar.s486211, PMID: 40060705 PMC11890310

